# Supplementary material for: HIVprotI: an integrated web based platform for prediction and design of HIV proteins inhibitors
Source: J Cheminform. 2018 Mar 9;10:12. doi: 10.1186/s13321-018-0266-y (PMC5845081; doi:10.1186/s13321-018-0266-y)
Supplement: Supplementary file 2 — Additional file 2. Source code of HIVProtI web server. [file 13321_2018_266_MOESM2_ESM.zip › HIVprotI_Source-code/hivprotI/weka-3-6-6/doc/weka/gui/CheckBoxList.CheckBoxListModel.html]

CheckBoxList.CheckBoxListModel


---


|  |  |  |
| --- | --- | --- |
| **Overview** \| **Package** \| **Class** \| **Tree** \| **Deprecated** \| **Index** \| **Help** \| **Weka's home** | |  |
| **PREV CLASS**   **NEXT CLASS** | **FRAMES**    **NO FRAMES**     **All Classes** |  |
| SUMMARY: NESTED \| FIELD \| CONSTR \| METHOD | DETAIL: FIELD \| CONSTR \| METHOD |  |


---


## weka.gui Class CheckBoxList.CheckBoxListModel

```
java.lang.Object
  javax.swing.AbstractListModel
      javax.swing.DefaultListModel
          weka.gui.CheckBoxList.CheckBoxListModel
```

**All Implemented Interfaces:**: java.io.Serializable, javax.swing.ListModel

**Enclosing class:**: CheckBoxList

---

``` public class CheckBoxList.CheckBoxListModel extends javax.swing.DefaultListModel ```

A specialized model.

**Version:**
:   $Revision: 7059 $

**Author:**
:   fracpete (fracpete at waikato dot ac dot nz)

**See Also:**: Serialized Form

---

**Constructor Summary**

`CheckBoxList.CheckBoxListModel()`
  
          initializes the model with no data.

`CheckBoxList.CheckBoxListModel(java.lang.Object[] listData)`
  
          Constructs a CheckBoxListModel from an array of objects and then applies
setModel to it.

`CheckBoxList.CheckBoxListModel(java.util.Vector listData)`
  
          Constructs a CheckBoxListModel from a Vector and then applies setModel
to it.


| **Method Summary** | |
| --- | --- |
| `void` | `add(int index, java.lang.Object element)`             Inserts the specified element at the specified position in this list. |
| `void` | `addElement(java.lang.Object obj)`             Adds the specified component to the end of this list. |
| `boolean` | `contains(java.lang.Object elem)`             Tests whether the specified object is a component in this list. |
| `void` | `copyInto(java.lang.Object[] anArray)`             Copies the components of this list into the specified array. |
| `java.lang.Object` | `elementAt(int index)`             Returns the component at the specified index. |
| `java.lang.Object` | `firstElement()`             Returns the first component of this list. |
| `java.lang.Object` | `get(int index)`             Returns the element at the specified position in this list. |
| `boolean` | `getChecked(int index)`             returns the checked state of the element at the given index |
| `java.lang.Object` | `getElementAt(int index)`             Returns the component at the specified index. |
| `int` | `indexOf(java.lang.Object elem)`             Searches for the first occurrence of elem. |
| `int` | `indexOf(java.lang.Object elem, int index)`             Searches for the first occurrence of elem, beginning the search at index. |
| `void` | `insertElementAt(java.lang.Object obj, int index)`             Inserts the specified object as a component in this list at the specified index. |
| `java.lang.Object` | `lastElement()`             Returns the last component of the list. |
| `int` | `lastIndexOf(java.lang.Object elem)`             Returns the index of the last occurrence of elem. |
| `int` | `lastIndexOf(java.lang.Object elem, int index)`             Searches backwards for elem, starting from the specified index, and returns an index to it. |
| `java.lang.Object` | `remove(int index)`             Removes the element at the specified position in this list. |
| `boolean` | `removeElement(java.lang.Object obj)`             Removes the first (lowest-indexed) occurrence of the argument from this list. |
| `java.lang.Object` | `set(int index, java.lang.Object element)`             Replaces the element at the specified position in this list with the specified element. |
| `void` | `setChecked(int index, boolean checked)`             sets the checked state of the element at the given index |
| `void` | `setElementAt(java.lang.Object obj, int index)`             Sets the component at the specified index of this list to be the specified object. |
| `java.lang.Object[]` | `toArray()`             Returns an array containing all of the elements in this list in the correct order. |

**Methods inherited from class javax.swing.DefaultListModel**

`capacity, clear, elements, ensureCapacity, getSize, isEmpty, removeAllElements, removeElementAt, removeRange, setSize, size, toString, trimToSize`

**Methods inherited from class javax.swing.AbstractListModel**

`addListDataListener, getListDataListeners, getListeners, removeListDataListener`

**Methods inherited from class java.lang.Object**

`equals, getClass, hashCode, notify, notifyAll, wait, wait, wait`

**Constructor Detail**

### CheckBoxList.CheckBoxListModel

```
public CheckBoxList.CheckBoxListModel()
```

:   initializes the model with no data.

---


### CheckBoxList.CheckBoxListModel

```
public CheckBoxList.CheckBoxListModel(java.lang.Object[] listData)
```

:   Constructs a CheckBoxListModel from an array of objects and then applies
    setModel to it.

    **Parameters:**: `listData` - the data to use

---


### CheckBoxList.CheckBoxListModel

```
public CheckBoxList.CheckBoxListModel(java.util.Vector listData)
```

:   Constructs a CheckBoxListModel from a Vector and then applies setModel
    to it.


**Method Detail**

### add

```
public void add(int index,
                java.lang.Object element)
```

:   Inserts the specified element at the specified position in this list.

    :   **Overrides:**: `add` in class `javax.swing.DefaultListModel`
    :   **Parameters:**: `index` - index at which the specified element is to be inserted: `element` - element to be inserted

---


### addElement

```
public void addElement(java.lang.Object obj)
```

:   Adds the specified component to the end of this list.

    :   **Overrides:**: `addElement` in class `javax.swing.DefaultListModel`
    :   **Parameters:**: `obj` - the component to be added

---


### contains

```
public boolean contains(java.lang.Object elem)
```

:   Tests whether the specified object is a component in this list.

    :   **Overrides:**: `contains` in class `javax.swing.DefaultListModel`
    :   **Parameters:**: `elem` - the element to check **Returns:**: true if the element is in the list

---


### copyInto

```
public void copyInto(java.lang.Object[] anArray)
```

:   Copies the components of this list into the specified array.

    :   **Overrides:**: `copyInto` in class `javax.swing.DefaultListModel`
    :   **Parameters:**: `anArray` - the array into which the components get copied **Throws:**: `java.lang.IndexOutOfBoundsException` - if the array is not big enough

---


### elementAt

```
public java.lang.Object elementAt(int index)
```

:   Returns the component at the specified index. Throws an
    ArrayIndexOutOfBoundsException if the index is negative or not less
    than the size of the list.

    :   **Overrides:**: `elementAt` in class `javax.swing.DefaultListModel`
    :   **Parameters:**: `index` - an index into this list **Returns:**: the component at the specified index **Throws:**: `java.lang.ArrayIndexOutOfBoundsException`

---


### firstElement

```
public java.lang.Object firstElement()
```

:   Returns the first component of this list. Throws a
    NoSuchElementException if this vector has no components.

    :   **Overrides:**: `firstElement` in class `javax.swing.DefaultListModel`
    :   **Returns:**: the first component of this list **Throws:**: `NoSuchElementException`

---


### get

```
public java.lang.Object get(int index)
```

:   Returns the element at the specified position in this list.

    :   **Overrides:**: `get` in class `javax.swing.DefaultListModel`
    :   **Parameters:**: `index` - of element to return **Throws:**: `java.lang.ArrayIndexOutOfBoundsException`

---


### getElementAt

```
public java.lang.Object getElementAt(int index)
```

:   Returns the component at the specified index.

    :   **Specified by:**: `getElementAt` in interface `javax.swing.ListModel` **Overrides:**: `getElementAt` in class `javax.swing.DefaultListModel`
    :   **Parameters:**: `index` - an index into this list **Returns:**: the component at the specified index **Throws:**: `java.lang.ArrayIndexOutOfBoundsException`

---


### indexOf

```
public int indexOf(java.lang.Object elem)
```

:   Searches for the first occurrence of elem.

    :   **Overrides:**: `indexOf` in class `javax.swing.DefaultListModel`
    :   **Parameters:**: `elem` - an object **Returns:**: the index of the first occurrence of the argument in this list; returns -1 if the object is not found

---


### indexOf

```
public int indexOf(java.lang.Object elem,
                   int index)
```

:   Searches for the first occurrence of elem, beginning the search at index.

    :   **Overrides:**: `indexOf` in class `javax.swing.DefaultListModel`
    :   **Parameters:**: `elem` - the desired component: `index` - the index from which to begin searching **Returns:**: the index where the first occurrence of elem is found after index; returns -1 if the elem is not found in the list

---


### insertElementAt

```
public void insertElementAt(java.lang.Object obj,
                            int index)
```

:   Inserts the specified object as a component in this list at the
    specified index.

    :   **Overrides:**: `insertElementAt` in class `javax.swing.DefaultListModel`
    :   **Parameters:**: `obj` - the component to insert: `index` - where to insert the new component **Throws:**: `java.lang.ArrayIndexOutOfBoundsException`

---


### lastElement

```
public java.lang.Object lastElement()
```

:   Returns the last component of the list. Throws a NoSuchElementException
    if this vector has no components.

    :   **Overrides:**: `lastElement` in class `javax.swing.DefaultListModel`
    :   **Returns:**: the last component of the list **Throws:**: `NoSuchElementException`

---


### lastIndexOf

```
public int lastIndexOf(java.lang.Object elem)
```

:   Returns the index of the last occurrence of elem.

    :   **Overrides:**: `lastIndexOf` in class `javax.swing.DefaultListModel`
    :   **Parameters:**: `elem` - the desired component **Returns:**: the index of the last occurrence of elem in the list; returns -1 if the object is not found

---


### lastIndexOf

```
public int lastIndexOf(java.lang.Object elem,
                       int index)
```

:   Searches backwards for elem, starting from the specified index,
    and returns an index to it.

    :   **Overrides:**: `lastIndexOf` in class `javax.swing.DefaultListModel`
    :   **Parameters:**: `elem` - the desired component: `index` - the index to start searching from **Returns:**: the index of the last occurrence of the elem in this list at position less than index; returns -1 if the object is not found

---


### remove

```
public java.lang.Object remove(int index)
```

:   Removes the element at the specified position in this list. Returns the
    element that was removed from the list.

    :   **Overrides:**: `remove` in class `javax.swing.DefaultListModel`
    :   **Parameters:**: `index` - the index of the element to removed **Throws:**: `java.lang.ArrayIndexOutOfBoundsException`

---


### removeElement

```
public boolean removeElement(java.lang.Object obj)
```

:   Removes the first (lowest-indexed) occurrence of the argument from this
    list.

    :   **Overrides:**: `removeElement` in class `javax.swing.DefaultListModel`
    :   **Parameters:**: `obj` - the component to be removed **Returns:**: true if the argument was a component of this list; false otherwise

---


### set

```
public java.lang.Object set(int index,
                            java.lang.Object element)
```

:   Replaces the element at the specified position in this list with the
    specified element.

    :   **Overrides:**: `set` in class `javax.swing.DefaultListModel`
    :   **Parameters:**: `index` - index of element to replace: `element` - element to be stored at the specified position **Throws:**: `java.lang.ArrayIndexOutOfBoundsException`

---


### setElementAt

```
public void setElementAt(java.lang.Object obj,
                         int index)
```

:   Sets the component at the specified index of this list to be the
    specified object. The previous component at that position is discarded.

    :   **Overrides:**: `setElementAt` in class `javax.swing.DefaultListModel`
    :   **Parameters:**: `obj` - what the component is to be set to: `index` - the specified index **Throws:**: `java.lang.ArrayIndexOutOfBoundsException`

---


### toArray

```
public java.lang.Object[] toArray()
```

:   Returns an array containing all of the elements in this list in the
    correct order.

    :   **Overrides:**: `toArray` in class `javax.swing.DefaultListModel`
    :   **Returns:**: an array containing the elements of the list

---


### getChecked

```
public boolean getChecked(int index)
```

:   returns the checked state of the element at the given index

    :   **Parameters:**: `index` - the index of the element to return the checked state for **Returns:**: the checked state of the specifed element

---


### setChecked

```
public void setChecked(int index,
                       boolean checked)
```

:   sets the checked state of the element at the given index

    :   **Parameters:**: `index` - the index of the element to set the checked state for: `checked` - the new checked state


---


|  |  |  |
| --- | --- | --- |
| **Overview** \| **Package** \| **Class** \| **Tree** \| **Deprecated** \| **Index** \| **Help** \| **Weka's home** | |  |
| **PREV CLASS**   **NEXT CLASS** | **FRAMES**    **NO FRAMES**     **All Classes** |  |
| SUMMARY: NESTED \| FIELD \| CONSTR \| METHOD | DETAIL: FIELD \| CONSTR \| METHOD |  |


---
